# Emerging lasers and light-based therapies in the management of acne: a review

**DOI:** 10.1007/s10103-024-04196-8

**Published:** 2024-09-28

**Authors:** Philippe Jean-Pierre, Lea Tordjman, Arjun Ghodasara, Chika Nwosu, Keyvan Nouri

**Affiliations:** https://ror.org/02dgjyy92 grid.26790.3a 0000 0004 1936 8606 Dr. Phillip Frost Department of Dermatology and Cutaneous Surgery, Miller School of Medicine, University of Miami, 1600 NW 10th Ave., Miami, FL 33136 USA

**Keywords:** Acne vulgaris, Light therapy, Laser

## Abstract

Acne vulgaris, commonly known as acne, is the most prevalent skin disorder affecting mainly adolescents and young adults, though it can affect people of all ages, making it the most common complaint by patients presenting to a dermatologist. The overactivity of sebaceous glands primarily drives this skin condition due to androgen influence and the presence of *Cutibacterium acnes* bacteria. Although typically not directly harmful to patient health, acne can be a highly debilitating disease for patients, affecting their self-image and psychosocial well-being. Standard treatments include topical retinoids, benzoyl peroxide, topical antibiotics, and, for more severe cases, systemic antibiotics or isotretinoin, which require prolonged periods of compliance. All these pharmacologic treatments have a risk of side effects ranging from mild ones, like skin irritation and dryness, to severe ones, like depression. Thus, there is a demand for exploring other treatment modalities in treating acne, and laser and light-based therapies have garnered significant interest. This review article will comprehensively assess emerging laser and other light-based therapies that have shown efficacy in treating acne, including the recently FDA-approved 1,726 nm laser.

## Introduction

Acne vulgaris, or acne, is a skin disorder triggered by *Cutibacterium acnes* (*C. acnes*) and circulating androgens in the sebaceous glands. Together, these factors can create inflammatory lesions of varying severity [1, 2]. Acne is most prevalent among adolescents at the start of puberty up to the early thirties, but it can affect patients of all ages [3, 4]. Pharmacologic treatment usually takes 2–3 months and varies based on the severity of the acne and the psychological impact the appearance of the acne has on the patient [5]. Typically, topical retinoids or benzoyl peroxide are first-line therapies for mild acne. In moderate cases, tetracycline oral antibiotics are often incorporated; in severe cases, isotretinoin is sometimes used [5].

Although acne treatment with the previously mentioned treatments is often successful when appropriately completed, these regimens require consistent, daily compliance. Many patients, especially younger age groups, have difficulty fully adhering to the regimen and thus cannot achieve complete acne clearance [6]. Furthermore, some of these pharmacologic treatment options have a risk of adverse events ranging from skin irritation to serious adverse events like teratogenicity and depression associated with isotretinoin [6]. Additionally, there have been concerns about increasingly antibiotic-resistant *C. acnes* species [7]. Thus, there is an increasing demand for novel therapies for acne that are effective and safe. Laser and light-based therapies have emerged as treatment modalities, and recent advancements and studies increasingly show evidence that these can be highly effective in treating acne [8]. This study reviews the latest emerging laser and light-based treatments that have shown efficacy in treating acne.

## Light therapy in the management of acne

### Blue Light

Blue light (BL), defined by wavelengths within the 415–545 nm range, achieves a skin penetration of approximately 0.3 mm. BL therapy for acne vulgaris harnesses the photodynamic effect to target *C. acnes*. This bacterium produces light-sensitive porphyrins (coproporphyrin III and protoporphyrin IX) with the most substantial absorption between 407 and 420 nm. It is postulated that when exposed to BL wavelengths, these porphyrins are activated and produce highly reactive singlet oxygen and free radicals, which damage the lipid walls of *C. acnes* and result in bacterial destruction [9, 10]. Narrow-band blue light has also been thought to reduce inflammation in keratinocytes by inhibiting the cytokine-induced production of IL-1α and ICAM-1, thereby mitigating inflammatory responses in the skin [11].

A 12-week randomized clinical trial (RCT) by Antoniou et al. evaluated the efficacy and safety of the KLOX BioPhotonic System, an LED BL device combined with specific photo-converter chromophores, for the treatment of moderate to severe acne vulgaris. Treatment sessions were conducted twice weekly for six weeks to one hemiface of each patient, while the contralateral hemiface served as the untreated control. Patients were followed up every two weeks for six additional weeks post-treatment. All participants received a skin cleanser and a non-comedogenic cream with SPF 50 ultraviolet protection for daily use over the entire face. Efficacy was assessed using the Investigator’s Global Assessment (IGA) scale and inflammatory acne lesion counts at weeks 6 and 12. Results demonstrated a reduction of at least two grades in IGA severity in 51.7% of patients at week 12, with even higher success rates observed in patients with severe acne. 81.6% of treated hemifaces showed a reduction of at least 40% in inflammatory lesion counts after 12 weeks. Safety assessments revealed no serious adverse events, and patients reported improved quality of life, including decreased acne-related pain after the 6-week treatment period [12].

In a prospective study by Ammad et al., the efficacy of intense narrow-band BL (415–425 nm) therapy was evaluated for treating 21 patients with mild to moderate facial acne. Patients underwent 14-minute treatment sessions twice weekly for four weeks. Assessment of acne severity using the Leeds Grading System demonstrated a significant improvement (*p* = 0.001), with reductions observed in both inflammatory (*p* = 0.001) and non-inflammatory lesion counts (*p* = 0.06). Disability, as measured by the Dermatology Life Quality Index (DLQI) at baseline versus post-treatment, was also significantly ameliorated (*p* = 0.001). Significant improvements were observed as measured by the Visual Analog Scale (VAS) scores from patients and investigators (*p* = 0.01 and *p* = 0.001, respectively). However, the colony counts of *C. acnes* did not show a significant decrease (*p* = 0.660) by the end of the treatment [13].

These findings were supported by Kawada et al., who evaluated the efficacy of narrow-band BL therapy (415–420 nm; ClearLight™, Lumenis, Tokyo) in thirty patients receiving treatment twice weekly for five weeks. Clinical assessments were performed at various intervals, with lesion counts and global improvement ratings recorded. BL therapy reduced inflammatory lesions, specifically comedones, papules, and pustules, by 45.5%, 59.3%, and 46.8% at three weeks, respectively, and by 57.8%, 69.3%, and 73.3% at five weeks. The investigator-assessed improvement rate was 77% by week 5, with 40% of patients showing marked improvement or clearance of acne lesions. Tolerance to the treatment was high, with only two patients experiencing mild skin dryness. In contrast to reports by Ammad et al., in vitro studies demonstrated a significant reduction in *C. acnes* after irradiation, supporting the bactericidal effects of the blue light source. However, patients with co-cultured methicillin-sensitive or methicillin-resistant Staphylococcus aureus did not respond well to the treatment [14].

A clinical trial by Liu et al. investigated the efficacy of portable LED BL and red light (RL) sources in treating mild to moderate acne vulgaris over one month. Twenty patients were randomly assigned to the BL or RL group, receiving treatment twice a week for four weeks, followed by a one-month follow-up period. Patients treated with BL experienced a significant reduction in inflammatory lesion count, with an average decrease from 19.2 to 5.5 after eight sessions (*p* < 0.05). Conversely, the RL group showed no significant improvement in lesion counts, with some patients experiencing worsening symptoms. Side effects were minimal, with few reports of skin dryness [15].

Kwon et al. performed a 20-week RCT investigating the efficacy of sequential non-ablative 1,450-nm diode laser (DL) followed by visible BL therapy versus BL alone in 24 patients with mild to moderate acne vulgaris. Treatment protocols involved three consecutive sessions at four-week intervals. The results revealed statistically significant reductions in inflammatory acne lesions following a combination of DL and BL therapy and BL-only treatments. The combination regimen exhibited a 62.3% decrease compared to 35.2% with BL alone at the 12-week follow-up (*p* < 0.05). While BL proved effective against inflammatory lesions, incorporating DL yielded synergistic benefits in reducing inflammation and targeting sebaceous gland activity [16].

### Red Light

In contrast to BL, red light (RL) possesses weaker energy levels and diminished efficacy in porphyrin activation. Its longer wavelength, ranging 600–650 nm, allows for 1–2 mm deeper skin penetration, increasing damage to the sebaceous glands and eliciting an anti-inflammatory response [17]. RL’s anti-inflammatory properties potentially modulate cytokine release from macrophages, promoting fibroblast proliferation, growth factor secretion, and orchestrating the inflammatory milieu needed for wound healing and tissue repair. RL has been proposed as a tool to treat acne vulgaris through these mechanisms [18].

In 2007, Na et al. conducted a split-face RCT comparing the efficacy of phototherapy via a portable RL device (Softlaser SL30, Beurer GmbH & Co., Ulm, Germany) on acne vulgaris. 30 subjects with mild to moderate acne underwent RL therapy twice daily for eight weeks. A significant reduction in both non-inflammatory lesions (59% reduction at Week 8, *p* < 0.005) and inflammatory lesions (66% reduction at Week 8, *p* < 0.005) compared to the untreated control side was found. Moreover, the total lesion count exhibited a decrease (55% reduction at Week 8, *p* < 0.005), alongside a significant improvement in VAS scores (*p* < 0.005) at Week 8, indicative of enhanced patient satisfaction [19].

In a study by Zane et al. investigating the efficacy of RL phototherapy in treating moderate acne vulgaris, 15 female participants with facial and truncal papular and pustular acne were enrolled. RL, delivered by a high-pressure metal halide lamp, was administered twice weekly for four weeks to the face at a fixed dose of 20 J/cm2. Lesions of the chest and upper back served as untreated controls. Clinical improvement was assessed using the Global Acne Grading System (GAGS) scores, revealing a significant reduction in median GAGS-face from 16.0 to 8.0 at the end of therapy (*p* < 0.05). At the three-month follow-up, improvement was sustained (median: 8.0, *p* < 0.05). Significant reductions in skin sebum (132.7 ± 19.9 µg/cm2 to 74.6 ± 21.0 µg/cm2) and trans-epidermal water loss (20.3 ± 7.7 g/m2 h to 9.1 ± 2.6 g/m2 h) were observed post-treatment (*p* < 0.05). Untreated lesions on the trunk did not exhibit similar improvement. Adverse effects were minimal, with transient burning sensations being the most reported [20].

The efficacy and tolerability of photodynamic therapy (PDT) using methyl aminolevulinate (MAL) combined with RL versus intense pulsed light (IPL) for the treatment of acne vulgaris were investigated by Hong et al. In this 8-week RCT, 20 patients with active acne lesions underwent split-face PDT treatments with MAL plus RL (34 mW/cm2, Aktilite CL 128 device) on one side and MAL plus IPL on the other. Patients received three treatment sessions at 2-week intervals and were followed up until four weeks post-treatment. RL had a significantly lower acne grade at the second visit than IPL (3.1 vs. 3.4, *P* = 0.046). However, no significant difference in acne grade improvement between the two sides was observed after the third treatment at four weeks. The study demonstrated that MAL-PDT with both RL and IPL is effective and safe for acne treatment, with RL demonstrating a faster response time. Initial adverse effects, including pain, erythema, and edema, observed in Asian patients prompted the reduction of the total dose of RL from 37 J/cm2 to 22 J/cm2. This dose adjustment was implemented to enhance tolerability in Asian patients, highlighting the importance of personalized treatment parameters, including light intensity, based on ethnic variations in skin characteristics and sensitivity to light [21].

A 2000 study by Papagergiou et al. examined the efficacy of combining BL (415 nm) and RL (660 nm) therapy versus BL therapy with benzoyl peroxide (BP) for treating acne vulgaris. 107 patients with mild to moderate acne were randomized into three groups: (a) BL + RL, (b) blue light, (c) BP, and (d) a control group using white light. Significant improvements were observed in inflammatory lesions and comedones with BL + RL therapy compared to BL or BP. The combined therapy resulted in a final mean improvement of 76% in inflammatory lesions and 58% in comedones. This suggests that BL and RL may synergistically improve acne by combining their antibacterial and anti-inflammatory properties [22].

A split-face trial by Slutsky-Bank et al. compared the efficacy and tolerability of daylight photodynamic therapy (DL-PDT) versus conventional red-light photodynamic therapy (C-PDT) for acne vulgaris. Fifteen patients underwent four treatment sessions at 3-week intervals, with half of the face treated with DL-PDT and the other half with C-PDT. Results revealed that DL-PDT was as effective as C-PDT in reducing inflammatory and non-inflammatory lesions, with statistically significant reductions observed in comedones (*p* < 0.05), papules (*p* < 0.01), and pustules (*p* < 0.05) on both sides of the face compared to baseline values. Notably, DL-PDT demonstrated a more significant reduction in pustular lesions than C-PDT (*p* = 0.023). Patients reported significantly lower pain levels during DL-PDT compared to C-PDT (*p* < 0.001), with a shorter downtime duration observed for DL-PDT (1.4 vs. 4.1 days, *p* = 0.001). No patient expressed a preference for one treatment over the other. These results suggested that DL-PDT may be a viable alternative to C-PDT for acne vulgaris treatment. It offers milder side effects, shorter downtime, and better accessibility due to its simplicity and reduced need for additional equipment [23].

### Intense pulsed light (IPL)

IPL has emerged as a modality for addressing acne vulgaris, offering a versatile approach through its emission of polychromatic light spanning 400–1200 nm wavelengths. IPL lamps emit high-intensity light characterized by their polychromatic, incoherent, and diffused nature, rendering them adaptable to a broad spectrum of wavelengths. This facilitates precise parameter selection tailored to individual skin types and dermatological concerns, including erythema, acne, and discoloration [24, 25]. IPL’s therapeutic mechanism hinges on selective photothermolysis, whereby light penetrates the skin to target specific chromophores [26]. By leveraging different absorption capacities of various structures within the skin, IPL can selectively heat and coagulate target tissues, inducing destruction through thermocoagulation [27]. IPL’s mechanism in treating acne involves selective photothermolysis, targeting key chromophores in the skin, including hemoglobin, melanin, water, and porphyrins, a key metabolite of *C. acnes*. It is suggested that both bactericidal and anti-inflammatory effects be exerted while suppressing sebaceous gland activity and coagulating capillaries [28].

In a 2021 trial by Li et al., IPL combined with isotretinoin (0.5–0.75 mg/kg/day) was compared to isotretinoin alone in treating 47 Chinese participants with facial acne vulgaris graded 2–4 on the Global Evaluation Acne (GEA) scale. The IPL treatment (420 nm cutoff filter) consisted of bi-weekly sessions for four weeks. Both groups received topical adapalene 0.1% gel and fusidic acid 2% cream. Clinical evaluation at week 12 showed a significant reduction in GEA grade and total lesions (79.2% vs. 65.2% in study and control groups, respectively) and particularly inflammatory lesions (79.2% vs. 56.5% in study and control groups, respectively) (*p* < 0.05). Mild to moderate pain during IPL treatment was reported in the study group, with no severe adverse events observed. As assessed by the DLQI and VAS, patient satisfaction was significantly higher in the study group (*p* < 0.05 and *p* < 0.01, respectively). Follow-up at two months post-treatment demonstrated a lower relapse rate or appearance of new lesions in the study group compared to the control group (*p* < 0.05) [29].

Qu et al. investigated the efficacy and safety of combining minocycline with IPL versus minocycline alone for treating moderate to severe facial acne vulgaris in 40 participants. All patients received minocycline (100 mg) daily for eight weeks. The IPL/minocycline combination group underwent 3 IPL treatments at weeks 0, 4, and 8. Results revealed significant improvements in both groups’ overall inflammatory lesion counts and severity scores when comparing baseline to week 16 (*p* < 0.02). However, the combination therapy group exhibited significantly greater improvements in inflammatory lesion counts (*p* < 0.04) and IGA scores (*p* ≤ 0.02) at weeks 4, 8, and 16. IPL treatment also effectively reduced the erythema index, an effect not observed with minocycline alone. Safety assessments showed no severe adverse effects in either group. Their results suggested that combining minocycline with IPL therapy using an acne filter spectrum of 400–600/800–1200 nm provides enhanced efficacy in reducing inflammatory lesions and severity of acne vulgaris, with a favorable safety profile [30].

A study by El-Latif et al. investigated the efficacy of IPL versus benzoyl peroxide (BP) in treating inflammatory acne lesions. 50 patients with Fitzpatrick skin phototype IV were randomly assigned to two groups. One group received BP 5% gel applied once daily, while the other underwent five sessions of IPL treatment (530-nm filter, 35 Joules/cm2 energy fluence, and 35 ms pulse duration) over five weeks. Both groups experienced statistically significant reductions in inflammatory lesion counts. In the BP group, the mean reduction in lesion count after the third session was 57.8% ± 20.4%, increasing to 69.4% ± 22.4% after the fifth session. Similarly, the IPL group showed a mean reduction of 42.4% ± 23.9% after the third session, increasing to 61.6% ± 26.1% after the fifth session. While both treatments demonstrated efficacy, BP yielded slightly better results than IPL, especially at the study’s midpoint (*p* < 0.05). While BP showed slightly superior efficacy, IPL treatment exhibited better tolerability with fewer side effects compared to BP, causing notable skin irritation [31].

Similarly, Chang et al. assessed the efficacy of IPL (530- to 750-nm filter) versus benzoyl peroxide (BP) for treating inflammatory acne. 30 female patients with mild-to-moderate acne underwent split-face IPL and BP gel treatments. Lesion counts revealed no significant difference between IPL-treated and BP-treated sides for mean inflammatory lesion counts. However, 63% of red macules on the IPL-treated side showed improvement compared to 33% on the BP-treated side (*p* < 0.05). Improvements in pigmentation and skin tone were more pronounced on the IPL-treated side. Although IPL treatment did not significantly affect inflammatory lesion counts, it effectively improved red macules, irregular pigmentation, and skin tone [32].

### Photopneumatic therapy

Photopneumatic therapy utilizes vacuum pressure in combination with IPL or lasers to treat acne. The vacuum draws structures in the dermis closer to the skin’s surface, which can subsequently be targeted more effectively by IPL. Shamban et al. retrospectively examined clinical data from 56 patients with varying degrees of acne who underwent 2 to 4 treatments using a portable photopneumatic device called Aesthera PPx. This device delivers broadband light (400 to 1200 nm) to the acne-affected areas via a handpiece. According to physician-evaluated clearance rates, mean clearance rates for acne ranged from 50% after a single treatment to 90% after four treatments in patients with mild-to-severe acne [33]. Gold et al. supported these findings in a study involving 11 subjects with mild to moderate acne, where all participants received four photopneumatic treatments spaced at 3-week intervals. Results demonstrated significant reductions in both inflammatory (*P* = 0.0137) and non-inflammatory (*P* = 0.0383) lesion counts at the 3-month mark [34].

Wanitphakdeedecha conducted a study to evaluate the efficacy and safety of photopneumatic therapy. The study involved 20 adults with mild to severe facial acne who underwent four consecutive treatments at 2-week intervals using a combined photopneumatic device. The results showed that most patients observed a slight decrease in acne lesions and overall clinical improvement; however, those with severe acne showed the most significant clinical enhancement. Mild side effects, such as temporary redness and occasional purpura, were limited [35].

Politi et al. performed a clinical trial evaluating the efficacy of incorporating cooling photopneumatic therapy with the 1540-nm Erbium: glass laser (later discussed in this review) to treat cases of mild-to-moderate acne. The trial recruited 12 patients aged 17 to 27 years who received four to six laser treatments (the number of treatments was determined by clinical improvement) in 2-week intervals. All patients completed the regimen and demonstrated progressive improvement throughout the treatments, with a reduction in both inflammatory and non-inflammatory acne lesions at 1 and 3-month follow-up from the last treatment. The only reported adverse events were mild erythema and edema that gradually decreased, and the edema was completely resolved at 1 and 3-month follow-ups [36].

## Laser therapy in the management of acne

### Novel 1,726 nm laser

In 2022, the FDA approved AviClear^™^ and Accure^™^, which combine a 1,726 nm laser with a contact-cooling sapphire window to treat mild, moderate, and severe acne. The 1,726 nm laser works through a process known as selective photothermolysis, which involves the laser’s wavelength being preferentially absorbed by sebaceous glands of the skin. Unlike many other lasers that also treat acne, selective thermolysis offers notable benefits, sparing the epidermis and preventing adverse events like skin damage and pain [37]. The sapphire cooling window is used to cool the skin before the laser application and immediately cool the skin after the laser application. This further helps protect the integrity and minimize damage to the skin by preventing heat from being delivered to tissues adjacent to the sebaceous glands [38].

Alexiades et al. performed a prospective trial in which 104 subjects with moderate-to-severe facial acne were enrolled, with Fitzpatrick skin types II-VI being represented. 89 subjects completed the trial involving three laser treatments between 3-week intervals. The proportion of individuals with skin clear or nearly clear of acne rose from 0% at the start to 9%, 36.0%, and 41.8% at the 4-week, 12-week, and 26-week follow-ups. No adverse events occurred from the protocol, and the treatment was well-tolerated for all subjects. The outcomes were consistent across individuals with different skin types [39].

Goldberg et al. performed a similar trial involving the 1,726 nm laser with contact cooling with 17 participants. The patients received three laser sessions up to seven weeks apart. All subjects tolerated the treatments well, with no serious adverse events. Compared to initial measurements, there was a statistically significant decrease in ILC (inflammatory lesion counts) of 52–56% over 4–12 after the treatment was complete. Follow-ups done 24 months after treatment completion found that patients had a 97% reduction in ILC. The patients’ subjective assessment found that 71% were highly satisfied with the treatment [40]. This is worth highlighting, as few studies involving lasers and light-based studies have shown long-term efficacy at a 2-year mark like in this trial.

Further studies are needed to understand better the effectiveness and risks of adverse events from the 1,726 nm laser; however, preliminary studies show promising results that it can be highly effective in treating active acne lesions with a low side effect profile and long-term efficacy.

### Pulsed dye laser (PDL)

Choi et al. compared the therapeutic effects of PDL versus IPL in a split-face, single-blind, randomized controlled trial. Over 14 weeks, 17 patients with active inflammatory facial acne underwent treatments of PDL (585 nm, 40ms pulse duration, 8–10 J/cm2 energy) and IPL (530–750 nm, 2.5 ms pulse duration, 7.5–8.3 J/cm2 energy), each on one side of the face. Patients received four sessions at 2-week intervals, then followed for eight weeks post-treatment. Significant differences in lesion counts between IPL and PDL treatments were observed. IPL-treated sides exhibited reductions to 50% of baseline for inflammatory acne lesion counts after the first treatment, with a reduction to 34% after the last treatment. However, at eight weeks post-treatment, the counts rebounded to 45% of the baseline. PDL-treated sides showed slower but more sustained improvement, with counts at 64% of baseline after the first session and decreasing to 14% of baseline at eight weeks post-treatment. Both inflammatory and non-inflammatory acne lesions showed significantly better improvements following PDL treatments than IPL treatments (*p* < 0.05). Patient satisfaction scores tended to favor PDL, and histopathological examinations suggested a more pronounced decrease in inflammation and increased TGF-β expression with PDL treatment [41].

PDL has also been suggested to be a useful modality in conjunction with other traditional pharmacologic acne therapies. A study by Ibrahim et al. compared the effectiveness of oral isotretinoin alone (ISO) and oral isotretinoin plus PDL (ISO/PDL). The trial’s findings showed that although both groups showed significant improvements in acne severity, the ISO/PDL group had a significantly greater improvement at three and 6-month follow-ups. Furthermore, the ISO/PDL group experienced fewer adverse events and required a lower cumulative dose of isotretinoin to achieve successful results than the ISO group [42].

Lekakh et al. performed an RCT exploring the combined effects of PDL and salicylic acid therapy versus salicylic acid alone in treating moderate to severe acne. 18 adult patients completed the study, receiving three treatments in three-week intervals. Half of the patients received PDL and salicylic acid therapy, and the other half were treated with salicylic acid only. The results showed that both cohorts experienced significant improvement in acne at the end of the study; however, using the GEA scale, there was a statistically significant difference (*P* = 0.003) between the PDL + salicylic acid cohort at -1.61 and the salicylic acid-only cohort at -1.11, which indicates that combination therapy was more effective than salicylic acid monotherapy [43].

### Neodymium: yttrium-aluminum-garnet laser (nd: YAG)

The *Nd: YAG* 1320-nm laser is a popular non-ablative treatment with clinical benefits in skin rejuvenation and acne scarring. However, studies have also explored its benefits in treating acne. Orringer et al. conducted an RCT involving 46 patients with facial acne with at least a Leeds acne severity score of 2 out of 12. During the trial, participants underwent three non-ablative laser treatments every three weeks using a 1320-nm Nd: YAG laser applied to one half of their face. Patients underwent clinical evaluations at baseline and weeks 7 and 14, and the evaluations involved a systematic tally of papules, pustules, cysts, and comedones. ​​The laser therapy caused substantial discomfort − 74% of surveyed patients indicated at least moderate levels of discomfort, 24% of respondents characterized their discomfort as substantial, and 2% rated it as extreme. Despite pain experienced during administration, the treatments were generally well-tolerated, with only a few transient adverse events, including two cases of post-inflammatory hyperpigmentation and two instances of focal blisters. Overall, changes in lesion counts from the baseline did not show statistically significant differences, except for two instances. Firstly, there was a noteworthy decline of 27% in open comedones on the treated skin, in contrast to a 12% increase on the control skin. Secondly, a significantly lower lesion count for cysts was observed in the treated skin compared to the untreated skin at week 14. There were also no significant differences in sebum production when comparing treated and untreated skin [44].

In another study, Deng et al. conducted a trial in which 35 individuals with moderate to severe acne underwent fractional 1320 nm Nd: YAG laser treatment in a regimen consisting of 6 sessions spaced two weeks apart. The laser therapy was well tolerated, leading to a 57% decrease (*P* < 0.05) in inflammatory lesions and a 35% decrease (*P* < 0.05) in non-inflammatory lesions. Additionally, there was a notable 30% reduction (*P* < 0.05) in skin sebum levels following the treatment [45].

A 2020 randomized clinical trial by Monib et al. compared the efficacy of long-pulsed Nd: YAG laser (1064 nm, Synchro HP, DEKA) versus IPL (The Nova light system) therapy in treating inflammatory and non-inflammatory acne lesions. Thirty Fitzpatrick skin phototypes III-V patients were randomly assigned to the Nd: YAG or IPL groups. Both treatments were administered over three sessions at 2-week intervals. The Nd: YAG group demonstrated a significant reduction in total acne lesions after each session compared to the IPL group (*P* < 0.001). Additionally, the Nd: YAG group showed significantly greater improvement in non-inflammatory lesions than the IPL group (*P* = 0.0099). Subjective patient satisfaction scores also favored the Nd: YAG group, with significant differences observed after the third session (*P* = 0.011). Complications such as erythema and edema were comparable between the groups, but crust formation was significantly higher in the IPL group (*P* = 0.003). While both treatments were effective, Nd: YAG laser therapy demonstrated superior efficacy in reducing acne lesions, particularly non-inflammatory ones, with higher patient satisfaction and fewer complications [46].

### Erbium: glass laser (Er: glass)

Bogle et al. conducted a clinical study to explore the effectiveness of the 1,540-nm Er: glass laser in addressing inflammatory facial acne of moderate to severe intensity. The study involved 14 patients, who were treated four times at 2-week intervals. 6 of the patients remained in a double-arm study to investigate whether an extra treatment at the 6-month mark would extend the period without lesions. The treatment itself did not require anesthesia and was relatively painless. Six months after the initial treatment, investigators noted a 78% improvement in lesions without any changes in sebum levels. Patients who received treatment six months after the initial course held clearance at 80%, while patients without retreatment had 72% clearance nine months after the initial course [47].

In an entirely different study, the effectiveness of a 1,540-nm Er: glass laser on acne was measured after two years. The laser was combined with contact cooling, and patients involved in the study underwent four treatments at 4-week intervals. Out of the initial 25 patients involved in the study, three were lost to follow-up, and four underwent retreatment - resulting in 18 patients who still had acne lesions two years after the last treatment. The mean percent reduction in lesions was 71% at the 6-month follow-up, 79% at the 1-year follow-up, and 73% at the 2-year follow-up, with no notable side effects [48].

In a clinical trial by Liu et al., the effectiveness of a 1,550 nm Er: glass laser was tested on 45 acne patients aged between 19 and 34 years. The participants had a mean acne duration of 4.8 years and underwent four weekly sessions of laser treatment without any other concurrent acne treatments. The study found a consistent and significant decrease in acne lesions, with a peak reduction of 67.7% one month after the treatments and a notable reduction in the incidence of new lesions. Of the 45 patients, 37 were followed up long-term, and the mean percentage reduction in lesion count was 72% at six months, 79% at one year, and 75% at two years [49].

## Discussion

Acne vulgaris is the most prevalent skin condition globally and can cause significant psychological and physical distress to patients, underscoring the importance of further advancements in treatment approaches to enhance efficacy and minimize adverse events. The studies reviewed in our study have shown that laser and light-based therapies can be highly effective in treating acne, both as monotherapies and in combination with other therapies. Some caution must be considered when interpreting the findings of these results, particularly newer devices, as some lack robust clinical data at this time. Notably, a limitation is that many of the cited studies had relatively short follow-up periods, weakening the evidence, particularly when compared to other known therapies like isotretinoin, which has more robust studies and long-term studies assessing its efficacy and safety [50]. Additionally, it is important to highlight that skin irradiation with lasers may generate reactive oxygen species, which can trigger some reported side effects, such as erythema [51, 52]. More research is necessary to understand these treatments’ effectiveness and safety and studies with longer follow-up periods to understand the long-term efficacy better. Nonetheless, the current evidence in the literature points to laser and light-based therapies as effective therapies for acne that are worthy of further exploration.
